# Support Provided by Caregivers for Community-Dwelling Obesity Individuals: Focus on Elderly and Hispanics

**DOI:** 10.3390/healthcare11101442

**Published:** 2023-05-16

**Authors:** Tanisha Basu, Ujala Sehar, Ashley Selman, Arubala P. Reddy, P. Hemachandra Reddy

**Affiliations:** 1 Department of Internal Medicine, Texas Tech University Health Sciences Center, Lubbock, TX 79430, USA; tanisha.basu@ttuhsc.edu (T.B.);; 2 Nutritional Sciences Department, College of Human Sciences, Texas Tech University, Lubbock, TX 79409, USA; 3 Department of Speech, Language and Hearing Sciences, School Health Professions, Texas Tech University Health Sciences Center, Lubbock, TX 79430, USA; 4 Department of Public Health, School of Population and Public Health, Texas Tech University Health Sciences Center, Lubbock, TX 79430, USA; 5 Neurology, Departments of School of Medicine, Texas Tech University Health Sciences Center, Lubbock, TX 79430, USA

**Keywords:** obesity, dementia, Hispanics, non-Hispanic Caucasians, epigenetics, socio-demographics

## Abstract

Obesity is a chronic disease marked by the buildup of extra adipose tissue and a higher chance of developing concomitant illnesses such as heart disease, diabetes, high blood pressure, and some malignancies. Over the past few decades, there has been a global increase in the prevalence of obesity, which now affects around one-third of the world’s population. According to recent studies, a variety of factors, including genetics and biology as well as environmental, physiological, and psychosocial factors, may have a role in the development of obesity. The prevalence of obesity is often higher among Hispanic American groups than among White people in the U.S. Obesity is a widespread condition with a high risk of morbidity and death, and it is well-recognized that the prevalence of comorbidities rises with rising levels of obesity or body mass index. To combat the rising prevalence of obesity in the USA, especially among Hispanics, one of the fastest-growing racial/ethnic groups in the country, there is an urgent need for obesity therapies. The exact cause of this disparity is unclear, but some responsible factors are a lack of education, high unemployment rates, high levels of food insecurity, an unhealthy diet, inadequate access to physical activity resources, a lack of health insurance, and constricted access to culturally adequate healthcare. Additionally, managing obesity and giving needed/timely support to obese people is a difficult responsibility for medical professionals and their loved ones. The need for caregivers is increasing with the increased number of individuals with obesity, particularly Hispanics. Our article summarizes the status of obesity, focusing on Hispanic populations, and we also highlight specific factors that contribute to obesity, including genetics, epigenetics, biological, physiological, and psychosocial factors, medication and disease, environment, and socio-demographics. This article also reviews caregiver duties and challenges associated with caring for people with obesity.

## 1. Introduction 

Obesity is a chronic disease characterized by the accumulation of excess adipose tissue and an increased risk of comorbid health conditions (e.g., heart disease, diabetes, high blood pressure, and certain cancers) [1,2,3]. Body mass index (BMI) is the most widely used measure for the screening of obesity in clinical and community settings [4]. BMI is calculated as the weight in kilograms divided by the height in meters squared (kg/m^2^). The most commonly used classification of obesity is the one defined by the World Health Organization’s international adult classification of BMI (see Table 1) [5]. 

The global prevalence of obesity has increased three-fold between 1975 and 2016 [5]. According to the World Health Organization (WHO), an estimated 650 million adults were obese in 2016 [5]. Data from the 2015–2016 National Health and Nutrition Examination Survey (NHANES) indicates that the average prevalence of obesity among older adults in the United States (U.S.) was 39.8% and 35.7% among younger adults aged 20 to 39 years of age [6]. The global incidence of obesity and being overweight has doubled in the past four decades to a magnitude that approximately one-third (39%) of the world’s population is categorized as being overweight or obese [7].

Based on a report published by Chooi et al. in 2015, obesity levels are increasing in all age groups and both genders regardless of the geographical region, ethnicity, or socio-economic status; however, the prevalence of obesity has usually been greater in older individuals and women. This tendency is comparable across regions and nations despite the prevalence percentages of obesity and being overweight differing widely. For several advanced countries, the occurrence rates of obesity seem to have decreased in the past few years. For example, Asians have a greater percentage of body fat than Caucasians even with an identical BMI. Larger cardio-metabolic risk is linked with the localization of additional fat in the visceral adipose tissue and ectopic stores such as muscle and liver as well as in instances of a boosted fat to lean mass ratio (i.e., metabolic obesity at a normal weight). Merely counting on the BMI to evaluate its prevalence could obstruct upcoming interventions aimed at obesity prevention and control. The U.S. and Europe have the highest rate of overweight and obesity. The prevalence rates of overweight and obesity are similar among other countries. In the year 2015, Turkey and the U.S. had the highest prevalence of obesity and being overweight, while, France, and Colombia had the lowest [8]. According to the World Obesity Atlas 2023, the projected global prevalence of obesity will rise from 11% in 2010 to 18% in 2030. Similarly, in women, the rise will be from 14% to 20%, and in men, it will be 9% to 15%, respectively [9].

The global scope of overweight and obesity (OAO) is not entirely understood because most cases of OAO appear in high-income countries and do not have a known equivalent among other countries. Studies in 2019 on the estimated economic impact of OAO in 161 countries using a cost-of-illness approach was 2.19% of the worldwide gross domestic product (GDP) ranging on average from USD 20 per capita in Africa to USD 872 per capita in the United States (U.S.), and from USD 6 in low-income countries to USD 1110 in high-income countries. If the existing trends continue until 2060, the economic effects from OAO are expected to rise to 3.29% of global GDP with a major upsurge in lower-resource countries, with total economic costs growing four-fold in high-income countries. There is a need to tackle the global intensification of OAO to prevent the substantial jeopardy of the well-being of the worldwide population [10].

This article presents the status of obesity in the United States (U.S.) with a focus on Hispanics. This article highlights specific factors that contribute to obesity, including genetics and biology, physiological and psychosocial factors, medication and disease, environment and socio-demographics. By doing so, the article hopes to establish the need for research and assessment tools for caregivers of obesity, especially in the underserved Hispanic community. This article summarizes the caregiver duties and challenges associated with caring for people with obesity and suggests tools to alleviate their burden.

## 2. Obesity Rates According to Age, Sex, and Ethnic Groups: Focus on Hispanics

**Figure 1:**
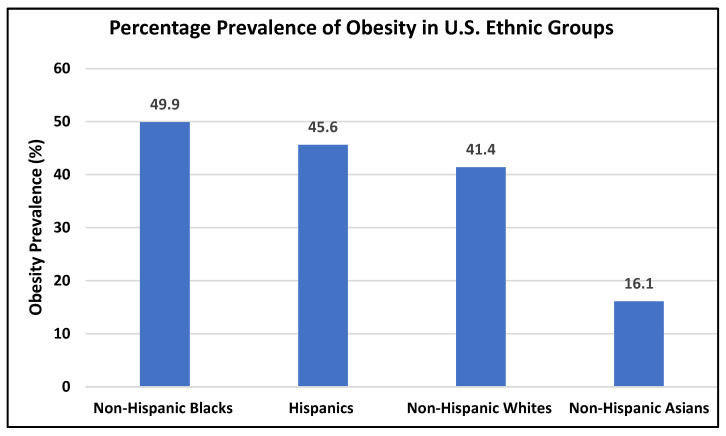
Percentage prevalence of obesity in U.S. ethnic groups based on the NHANES, 2021 [12].

During the past several decades, obesity rates continually rose at an unprecedented level. According to the 2021 National Health and Nutrition Examination Survey (NHANES), obesity rates in the United States (U.S.) increased from 30.5% in 1999 to 2000 and 41.9% in 2017 to 2022, and severe obesity rates increased from 4.7% to 9.2%, respectively. This increase is reflected across all age, sex, and ethnic groups; however, certain minority groups are more affected than others. According to the Centers for Disease Control (CDC), obesity is most prevalent among non-Hispanic and Black adults (49.9%), followed by Hispanic adults (45.6%), non-Hispanic Caucasian adults (41.4%), and non-Hispanic Asian adults (16.1%) (see Figure 1). These increases in obesity rates make obesity a major public health concern. In 2008 alone, the medical costs related to obesity in the U.S. was an estimated USD 147 billion [11,12].

Hispanic individuals within the United States (U.S.) have one of the highest rates of self-reported obesity (second only to non-Hispanic blacks). Twenty-seven states in the U.S. and the territory of Guam have obesity rates greater than 35% among the Hispanic population [12]. In comparison, only 10 states had greater than 35% obesity rates among non-Hispanic Caucasians. Although the exact cause of this disparity is unclear, various socio-economic and environmental disadvantages faced by the Hispanic community appear to be causal factors [13]. Some of these disadvantages include: (1) lack of education, (2) unemployment rates, (3) high levels of food insecurity, (4) poor diet quality, (5) inadequate access to physical activity resources, (6) lack of health insurance, and (7) poor access to culturally adequate healthcare [13]. Some studies indicate that obesity rates in the U.S. are directly associated with the socio-economic status (SES) and level of education [6]. Individuals with a lower SES are more likely to have obesity than those with a higher SES. The dietary habits of those in lower SES groups often involve consuming more unhealthy fats and simple carbohydrates, and less fruits, vegetables, and whole wheat bread. Additionally, lower-income neighborhoods are associated with limited access to exercise facilities and safety concerns, which contribute to higher rates of physical inactivity [14]. In a report published, it was noted that between 1984 and 2013, the educational disparity in regard to obesity remained consistent. Obesity prevalence grew from 17.46% to 36.16% among the lower education group, while it grew from 5.12% to 20.94% in those that had a college education [15].These statistics indicate that the prevalence of obesity-related comorbidities is also very high among U.S. Hispanics. Compared to 8% of non-Hispanic Caucasians, an estimated 17% of Hispanics are likely to develop type 2 diabetes in their lifetime. In addition, Hispanics are also more likely to develop prediabetes and have a 50% chance of developing diabetes as compared to 40% of the average U.S. adult population [16]. Hispanics also have an increased risk for developing cardiovascular diseases (CVD) potentially caused by obesity, diabetes, hypertension, psychological stress, and environmental hazards [17], which makes it the second leading cause of death among Hispanics [18].

Current studies measure the outcome of obesity as a fundamental reason of death as compared to non-communicable diseases (NCDs) in the U.S. as a causative root of mortality among Caucasian, Black, and Hispanic individuals. Diabetes caused a higher mortality rate for individuals with obesity than NCD-related conditions during the years 1999 to 2017 with Caucasians having the highest rate when matched with Black and Hispanic individuals [19]. Hispanics had lower mortality rates for cardiovascular disease (CVD) and diabetes. The average age of death was 57.3 years of age for individuals with obesity in all groups and those who died were 15.4 years younger than those who did not have obesity [20]. Although Hispanics in the U.S. have a higher frequency of diabetes and CVD, they also have the smallest mortality hazard ratio for obesity as an underlying cause of death when associated with CVD and cancer [19].

Food ingested that surpasses the body’s caloric needs is stored by the body as fat to be used in the future in the event of an energy deficit [3,21]. This evolutionary, physiological response prevents death by starvation and is often regulated or accompanied by various endocrine, neurological, and gastro-enteric processes. Optimal fat storage is critical to the functioning of physiological processes; however, an excess or deficit is harmful for health outcomes. It is important to note, moreover, that the rapid increase in obesity incidence cannot be attributed to genetics or the environment alone. Although not conclusive, one proposed mechanism states that the global obesogenic environment of hyper-caloric foods, sedentary lives, and environmental exposures work alongside the genetic programming of fat storage to result in the complex disease of obesity [22]. Moreover, to maintain the homeostasis of fat stores within the body, various endogenous mechanisms play a role. These are some of the key causative agents of obesity; therefore, it is important to note that when predisposed for these genetic and/or biological processes, an individual’s likelihood of becoming obese increases when exposed to contributing factors such as diet and exercise [3]. Studies show that several biomarkers are potential mediators in the pathogenesis of obesity and these include hormonal markers such as adipokines and cytokines, which are adipose tissue hormones and genetic markers such as those associated with leptin signaling [23].

## 3. Factors That Contribute to Obesity

Individuals develop obesity when their energy intake exceeds their energy expenditure, which results in a surplus of fat that is stored in adipocytes. The etiology of obesity, however, is multifactorial [3]. Current studies indicate that several factors may contribute to the development of obesity, such as genetics and biology, physiological and psychosocial factors (e.g., behavior), medication and disease, environment and socio-demographics [1,3,6].

### 3.1. Genetic, Epigenetic and Biological Factors of Obesity

Epigenetics is described as a hereditary and revocable phenomenon that affects gene expression without modifying the deoxyribonucleic acid (DNA) sequence. The mechanisms by which epigenetics occurs are DNA methylation, histone modifications, chromatin folding, and guiding noncoding miRNAs [24]. Epigenetic variations are influenced by environmental factors (e.g., nutrition, inflammation, physical activity, sex, and age) that regulate the environment–gene interaction and the reaction to lifestyle modifications.

The most important epigenetic mechanism related to obesity is DNA methylation. Methyl molecules covalently bind to cytosine residues within the DNA sequence during DNA methylation to classically decrease gene expression. Various studies associate DNA methylation with obesity and indicate that this process predicts obesity with a 70% confidence [25]. Other studies have used the Illumina 450 k array to identify various CpG sites that are associated with an increase in body mass index (BMI) in whole blood and DNA methylation and that were then later corroborated using adipose tissue samples [22]. Studies also show that in-utero and early life exposure to environmental modifications may cause genetic changes in the metabolic predisposition and cause obesity. Scientists found that pre-natal and post-natal nutrition also plays a crucial role in the development of obesity. One proposed mechanism is the “thrifty phenotype hypothesis” which suggests that poor fetal nutrition causes an increase in adiposity and decreased fat mobilization post-birth. Environmental triggers in early life, particularly in infancy, are linked to an increased risk in obesity and other chronic comorbidities. Children born in poverty, during famine, or who suffer malnutrition tend to develop a low tolerance to high-fat diets with an increased incidence in obesity and atherosclerosis [26].

Obesity is a disease that is accompanied by a very complex interplay of physiological pathways and mechanisms. Genetic alterations and mutations in these pathways are directly linked with the development of obesity. Some of these major pathways are mediated by signaling molecules that regulate feeding behaviors in the hypothalamus of the central nervous system (CNS). Leptin is a hormone secreted in white adipose tissues (WAT) and signals the hypothalamus about the fatty acid stores. Leptin plays important functional roles in fatty acid oxidation, female reproduction, and the immune response. In humans, leptin action takes place via the leptin receptor (OB-R). The absence of or mutations in the genes of this receptor can cause obesity. This type of obesity is treatable through exogenous leptin [27]. Leptin is the main human satiety hormone and works by suppressing appetite. Although individuals with obesity produce more leptin, they may not experience increased appetite suppression because people with greater adiposity develop leptin resistance [1,2]. Adiponectin, a cytokine secreted by white adipose tissue (WAT), can help reduce obesity by increasing insulin sensitivity, reducing inflammation, and speeding up fatty acid oxidation. Increased body fat stores, however, inhibit the production of adiponectin [2,3]. Insulin secreted by islet cells in the pancreas mediates glucose uptake and production and glycogen stores. Studies show that a glycogen buildup in the adipocytes are associated with insulin resistance and obesity. Other key CNS pathway molecules that regulate hunger and feeding include neuropeptide-y (NPY) and agouti related protein (AgRP), which are appetite stimulators, and proopiomelanocortin (POMC), which is an appetite inhibitor [2,27].

#### Focus on Hispanics

Scientists conducted a study that checked the saliva of 75 Hispanic children having the 17 CpG marker for maternal obesity for epigenetic markers [28]. The results indicated that the methylation of Cg1307483 (NRF1) was significantly associated with the emergence of childhood obesity at follow-up [28,29]. Another study conducted using a large Hispanic population linked maternal obesity or diabetes using differential DNA methylation in the epigenome of the fetus concerning genes (e.g., SLC2A9, HOOK2, APOE LTF, and DUSP22), which are associated with inflammatory regulatory pathways, signaling pathways, and the related symptoms of obesity and diabetes [30].

The SLC2A9 gene has been shown to be a major cardiac risk factor in Hispanics. The gene encodes GLUT-9 and an urate transporter, and the upregulation of it leads to hyperuricemia, and has also been correlated with the BMI, waist circumference, and the anti-oxidant status based upon the actions of different SNPs on the gene [31]. The GLUT-9 transporter functions to all the transport of various sugars (i.e., glucose, and fructose) [30]. Samples taken from diabetic mothers in a study completed by Stanirowski et al. revealed an increased expression in the GLUT-9 transporter [24,30,32]. Voruganti et al. found a significant association between the obesity-promoting SNP and SLC2A9 in the Mexican-American population, offering a potential epigenetic contribution to the increased incidence in obesity in the Hispanic population [31].

The Hook Microtubule Tethering Protein 2 (HOOK2) gene is another epigenetic contributor to increased rates in obesity within the Hispanic population. Rizzo et al. found HOOK2 to be hypomethylated in the region of CpG sites [30]. HOOK2 contributes to the pericentrosomal localization and formation of aggresomes, which are accumulations of misfolded proteins [33]. The apolipoprotein E (APOE) has a key role in the metabolism and transport of lipids and mitochondrial integrity [34]. The specific variants, rs439401 and rs11208712, were linked to a susceptibility to increased adiposity and inflammatory states, particularly in Hispanic populations [35].

Genetic mutations, congenital predisposition, hormonal irregularities, and inflammation are a few physiological issues that may predispose individuals to become obese. Presently, less than half of the United States (U.S.) population attain the daily recommendations for physical activity. Physical activity amenities are far less available and sedentary lifestyles are more predominant, which creates an environment favorable to reduced energy expenditure. The following psychosocial factors can also predispose someone to have obesity: depression, eating disorders, the stigma of having obesity, body image issues, behavioral issues, accessibility, affordability, and a lack of awareness of what constitutes a healthy diet [6,8].

### 3.2. Medication and Disease Factors

These common classes of medications are also known to contribute to weight gain: psychotropic medications, diabetic treatments, anti-hypertensive medications, steroid hormones, contraceptives, antihistamines, and protease inhibitors [36]. Research indicates that an infection may increase the risk of obesity [37]. Studies show that obesity in animals is caused by Adenovirus-36 (Ad-36) [38]. Research shows that people with obesity are more susceptible to being infected with Ad-36 [39]. Therefore, obesity can be considered a multifactorial disease with far reaching consequences regarding the health and well-being of individuals across all age groups.

Impaired energy balance is the main underlying factor that influences obesity; therefore, mechanisms that impair energy expenditure also play a causative role in the development and progression of obesity [1,3]. The energy requirement of an individual is based on their resting metabolic rate (RMR) which is the amount of energy required to maintain bodily functions at rest. The RMR combined with physical activity and thermogenesis creates the total energy expenditure. Certain endocrine conditions may impede energy metabolism and in turn cause obesity. These include hyperthyroidism, where a deficiency in the thyroid hormone causes a reduced metabolic rate, lethargy, and weight gain. Similarly, deficiencies in the growth hormone are related to weight gain as well potentially due to decreased thermogenesis [3]. Brown adipose tissues (BAT) consist of adipocytes, which are highly thermogenic due to an increased number of mitochondria. BATs are usually present in the supraclavicular, neck, and axillary regions and are associated with increased thermogenesis and energy expenditure [40]. BATs are inversely associated with obesity and its comorbidities.

#### Focus on Hispanics

Hispanic individuals have greater insulin resistance as compared to non-Hispanic Caucasians [41]. One proposed mechanism suggests high levels of acculturation stress may cause Hispanic individuals to develop physiological manifestations such as obesity. This model, referred to as the “allostatic load” hypothesizes that due to the various challenges of immigrant life, coupled with poor maternal and child nutrition, Hispanics develop the biological markers of obesity. Research indicates that Hispanics who live in the United States (U.S.) tend to gain four times more weight than recent immigrants do [42]. Moreover, some theories suggest that Hispanics tend to develop obesity because they are subject to a chronic state of elevated cortisol caused by exposure to stress [42]. Another key factor for the extremely high prevalence of obesity among Hispanics is the vicious cycle in which maternal obesity influences childhood obesity which in turn influences adulthood obesity [28,30].

### 3.3. Environmental and Socio-Demographic Factors

Environmental factors critically influence the development of the energy intake and energy expenditure balance, right from the pre-natal stages [43]. Many environmental factors that affect weight include physical, chemical, biological agents, urban housing and social conditions, transportation and pollution related to the proximity of influential agricultural or industrial environmental centers of activity. Access to schools, modes of transportation, the wide availability of technology such as smart-phones and video games, and presence of obesogens in the environment are a few conditions that have been proven to increase the risk in obesity among children [43,44]. Moreover, the presence of obesity seems to have a close correlation with individual- and population-based SES [44]. In developed countries, the obesity risk is inversely proportional to SES, and more prominently so among women. Alternatively, there has been a directly-proportional association between obesity and SES in developing countries [45]. In the U.S., individuals with food insecurity have 22% increased odds of developing obesity as opposed to those who were food secure through childhood [46]. Furthermore, socio-demographic features such as food availability, food deserts, nutrition knowledge, feeding behaviors, and cultural norms among others, often serve as obesogenic factors and/or barriers to obesity prevention/management [44,47].

#### Focus on Hispanics

Hispanics have a very high prevalence of obesity compared to non-Hispanic Whites and Asians, only second to non-Hispanic Blacks. While there is no conclusive evidence to elucidate the causes of this difference, some suggest that it is a combination of genetic and epigenetic factors along with differences in socio-cultural factors such as body image standards, SES and cultural-specific feeding behaviors [44]. Hispanics are more likely to live in poverty and to not earn a college degree as compared to non-Hispanic Whites and Asians. These characteristics of poor SES are associated with poor feeding behaviors such as eating calorie-dense foods, often from cheap, fast-food options and not engaging in physical activity unless as a means of employment. Moreover, the cost and availability of healthy foods, and unhealthy feeding behaviors are promoters of childhood weight gain among Hispanic youth [48]. When compared to their non-Hispanic White counterparts, Hispanics are usually hesitant to seek formal care due to various factors such as financial resources, a lack of awareness, a lack of culturally-competent caregiving facilities, language barriers, and a general fear of the healthcare system due to immigration or health insurance status [49,50].

### 3.4. Diet and Exercise Factors

Energy intake that surpasses energy expenditure leads to energy storage in the form of fats, an accumulation of which leads to obesity [3,4]. Although not a causative agent by itself, energy intake through diet is the single most powerful mediator of obesogenic factors. Energy consumption in the form of dietary intake is influenced by physiological processes such as hormone action, central nervous system (CNS) pathways, psychological factors such as stress and mental health, environmental factors such as access to grocery stores and fast-food restaurants, socio-economic status and food insecurity, and political factors such as nutrition policies [3,4,36]. A combination of some or all of these factors creates a conducive environment to trigger the epigenetic response surrounding obesity. Moreover, the style of eating in the United States (U.S.) greatly surpasses the dietary recommendations of caloric needs and macronutrients, is ridden with processed foods, and is characterized by large portions [1,2,3].

Energy expenditure, on the other hand, is mediated by non-exercise-related activity and exercise. The 2018 Physical Activity Guidelines for Americans recommend that all adults, including those with chronic conditions or disabilities, accumulate at least 150 to 300 min per week of moderately-intense physical activity [2]. To properly maintain weight, these guidelines recommend more than 300 min per week of moderately intense exercise combined with muscle-strengthening activities to maintain lean body mass. Currently, approximately one-fourth of U.S. adults meet physical activity guidelines. Prospective epidemiologic evidence shows that lower physical activity levels are associated with a higher risk of obesity; however, exercise only consists of a portion of energy expenditure, with most of the primary source being non-exercise-related day-to-day activities [1,3].

#### Focus on Hispanics

Because the Latino population is projected to increase from 18% of the total U.S. population to 29% by 2060, it is a critical health priority to improve their diet to prevent obesity and reduce the risk of obesity-related disease among Hispanic/Latinos. Research suggests that the diet quality among Hispanic/Latino adults is negatively-influenced by socio-demographic and socio-cultural factors, such as age, education, household income, and cultural beliefs. Acculturation is a well-researched socio-cultural factor affecting the dietary patterns of immigrant populations. Greater acculturation is associated with a higher intake of less healthy foods and a lower intake of healthy foods among Hispanic/Latino adults [51]. For example, the high fructose corn syrup-sweetened beverages available in the U.S. are associated with increased calorie consumption and potential weight gain [52]. Studies indicate that Hispanic men and women engage in less physical activity during leisure time than non-Hispanic Caucasians. Moreover, the research shows that sedentary behavior during leisure time was higher in Hispanics than in non-Hispanic Caucasians regardless of income levels [53].

## 4. Obesity Management Strategies

Obesity management is built on a foundation of comprehensive lifestyle medications, which include behavioral therapy, physical exercise, and diet. Clinicians can deliver these interventions and assist weight management in the primary care setting with the aid of new tools and treatment philosophies. The following are a few strategies for obesity prevention and management.

### 4.1. Lifestyle Interventions

**Figure 2:**
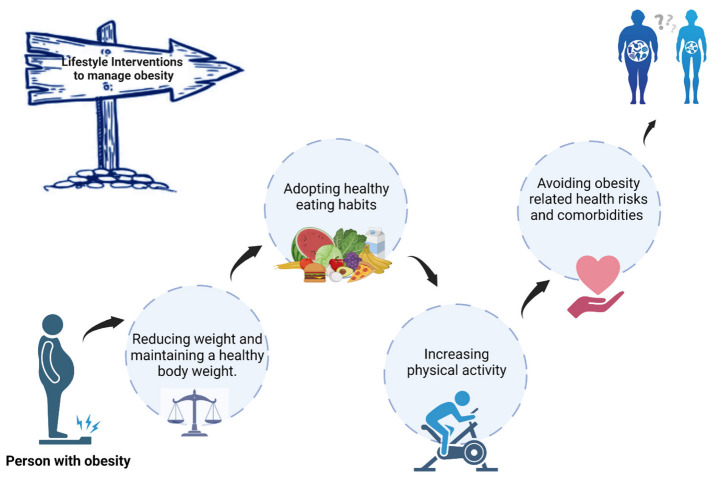
Lifestyle interventions that improve adherence to recommendations for maintaining healthy weight, adopting healthy eating habits, a reduced-calorie meal plan and increased physical exercise are recommended to patients who are overweight or obese.

Lifestyle interventions (e.g., diet and physical activity) is an essential part of all obesity treatment, refer to Figure 2. Calorie restriction alone (CR), also called continuous energy restriction (CER), is a major contributor to weight loss within these programs. CR combined with overall lifestyle intervention is the standard care prescribed in obesity treatment programs and can result in an average of 5% to 10% weight loss. To achieve successful weight loss and sustain it over time, studies suggest: (1) lifestyle interventions that change eating behaviors [3,39], and (2) a diet that reduces the overall energy intake, improves dietary quality, and increases energy expenditure. Additional therapeutics, such as cognitive behavioral therapy, pharmacotherapy, and even bariatric surgery may be required to successfully treat overweight and obese individuals [2,5,38].

Dietary interventions for weight loss come in various forms, but are all designed to maintain a hypocaloric state based on the: (1) manipulation of macronutrient content (i.e., low-fat, high-protein, low-carb diets), (2) a restriction of specific foods and/or food groups (i.e., gluten-free diet, paleo diet, vegetarian/vegan diet, and Mediterranean diet), and (3) a manipulation of eating times (i.e., fasting) [3].

### 4.2. Dietary Calorie Restriction

Calorie restriction, which is defined as a decrease in the caloric intake below ordinary ad libitum intake without malnutrition, has been shown to delay aging and lengthen the maximum life span in a variety of animal models, including yeasts and non-human primates [54]. Results from epidemiologic research also point to the possibility that caloric restriction can have positive impacts on the variables influencing the etiology of age-related chronic disease and human life expectancy. A standard, reduced-calorie diet which prescribes a reduction of 500–1000 kcal in daily energy consumption can yield a health weight loss of up to 2 lbs. per week. An energy-restricted diet is usually the first line of treatment for people who are overweight or with obesity who are thought to benefit from weight loss. Low-calorie diets, also known as low-energy diets, hypocaloric diets, or calorie restriction, are weight-loss techniques for people who are overweight or obese to enhance their metabolic health and lower the risk of illnesses linked to obesity [55]. The most crucial element in weight loss is an energy deficit; however, metabolic changes to reduce energy intake can also result in decreased energy expenditure. Long-term methods of creating an energy deficit are therefore required. A very low-calorie diet and meal replacement diets might be helpful in the short term if typical low-calorie diet plans do not work or when significant weight loss is required [56]. Very low-carbohydrate (ketogenic) diets are well-liked while being frequently dismissed as fad diets. Recent clinical investigations show they are superior to low-fat diets for encouraging rapid weight reduction and modifying metabolic syndrome symptoms. There is no doubt that a range of dietary interventions can help people lose weight, but the impacts on other areas of their health also need to be considered and thoroughly researched [57]. It has repeatedly been demonstrated that a calorie-restricted diet decreases lean body mass and bone mineral density at the hip as measured by dual-energy x-ray absorptiometry [58]. A diet restricted in calories alone improves the measurable outcomes of physical function and quality of life.

Obesity and insulin resistance are far more common in Hispanic children and adolescents than in non-Hispanic White youngsters. A randomized trial was conducted to study the effects of a low glycemic load or low-fat diet in Hispanic children and adults. When administered as part of a comprehensive culturally-appropriate weight-loss program, both a low glycemic load and low-fat diet reduced the BMI scores in study participants [59]. Latinos’ weight reduction needs to be addressed with culturally-appropriate methods. For instance, traditional Latino diets tend to consume more carbohydrates than the diets of Whites or African Americans, which may affect how well-liked low-carbohydrate diets are among Latinos [60]. Dietary acculturation has been observed in the Hispanic population in the U.S., and this diet change in the Hispanic population may be influenced by a variety of environment- and psychological-related dietary factors. It is crucial for health professionals to pinpoint the root causes of unhealthy eating habits and choose the best ways to address them. Obesity and diabetes, two diet-related health problems frequently observed in the Hispanic population, may be prevented or managed by recognizing and treating harmful dietary changes and habits [61]. According to the Dietary Guidelines 2020–2025, a heathy eating plan for healthy weight maintenance should include fruits, vegetables, whole grains, and fat-free or low-fat milk and milk products, a variety of protein foods and limit added sugars, sodium, trans fats and cholesterol [62]. The Food and Nutrition Board of the Institutes of Medicine recommends that 45–65% of daily energy comes from carbohydrates, 10–35% from proteins, and 20–35% from fats [63].

### 4.3. Physical Activity

Given that obesity is a global health concern, the implementation of accessible and feasible physical activity strategies is imperative now more than ever. Physical inactivity is a very well-known modifiable contributor to weight gain [64]. Studies have shown that following physical activity recommendations as part of a comprehensive lifestyle intervention regime may help reduce body weight, body fat, and visceral fat while maintaining muscle mass [64,65]. In the absence of a calorie-restricted diet, exercise that combines the elements of progressive resistance training and aerobic endurance training does not significantly reduce body weight, but it has good benefits on body composition, physical function, and quality of life in aging persons with obesity [65]. Moreover, studies have shown that physical activity may improve the outcomes of conditions such as CVD, type 2 diabetes, hypertension, osteoporosis, and certain forms of cancer, even in the absence of weight loss [66]. According to the CDC, 150 min/week of moderate-intensity exercise is required to maintain body weight, which needs to be adjusted according to calorie-deficit needs to achieve weight loss [67].

Research is limited on physical activity intervention studies in Hispanics, but it is noted that they have a lower incidence of meeting the guidelines [68]. Hispanic women, for example, have participated in fewer activities than men. Moreover, it was seen that acculturation reduced leisurely activity and increased work-related activities within this population [69]. Based on a review published from the Guide to Obesity Prevention in Latin America and the U.S. (GOL) parent study, out of 22 studies, more than half found a significant improvement in body weight or BMI in the short term in U.S. Latino adults who were overweight/obese. The review reported some limitations of the studies such as low recruitment due to participant concerns regarding immigration policies at the time, the recruitment of mostly female participants, and respondent bias due to self-reporting measures, among others. The review concluded that there was a huge gap in culturally-competent interventions that targeted obesity within the Hispanic population [70]. A huge contributor to this is the general under-representation of ethnic minorities in clinical research [71], particularly, the Hispanic population that has various socio-demographic and ethnocentric barriers that cause a mistrust of the U.S. healthcare system leading to a reluctance for participating in research [50]. Another systematic review reported that individual-based interventions in Latino adults in the U.S. reported an average of an 8.7 pound weight loss as compared to a 0.8 pound weight gain in the control groups [72].

### 4.4. Weight Management and Lifestyle Intervention for the Elderly

In 2019, 9% of the elderly population in the U.S. was made up of Hispanics. By 2030, the number of Hispanic elderly has been predicted to become almost twice as that in 2019 [73]. Obesity raises the risk of early disability and mortality and is linked to several chronic diseases, including osteoarthritis, cancer, hypertension, hyperlipidemia, diabetes, and sleep apnea [74]. Thus, older men and women who are overweight or obese at age 65 will spend more money on healthcare throughout the rest of their lives [75]. The co-occurrence of obesity and aging may have a significant impact on the socio-economic burden. According to the CDC, 76.9% of older males and 73.8% of older females are overweight and 36.4% of older males and 44.2% of older females are obese. The amount of adiposity increases with age and is the highest approximately at the age of 70 years. Among various potential contributors, some of the key factors that influence obesity with aging are a reduced RMR and, reduced energy expenditure due to physical inactivity and muscular atrophy (sarcopenia). Moreover, the hormonal action of testosterone, growth hormone, leptin, and thyroid hormone decline with age as well. Given the added detrimental impact of aging on the existing health effects of obesity, extra attention should be paid to older adults regarding weight management and lifestyle intervention programs. A complete understanding of the medical history of older adults, including any history of weight fluctuations, is crucial to a nutritional assessment [76]. Many older person’s health can be adversely affected by one or more chronic illnesses, which can affect eating habits and lead to poor health outcomes due to nutritional deficiencies. In the following section, the research on lifestyle interventions to treat obesity in older people is summarized.

Studies on weight loss drugs, however, are inconclusive in older people and there may be a possibility of drug side effects and interactions. Weight loss surgery appears to be successful with minimal risk among older adults [77].

Firstly, before calorie restriction is widely used in elderly individuals, there are several problems that need to be resolved. Secondly, it is unclear if the link between fat and unfavorable outcomes applies to senior persons as well. As we age, the relative risk linked to a higher BMI is decreased [78]. Even though a calorie-restricted diet is the first therapeutic option for the treatment of obesity in the general population, there are worries that this strategy may be hazardous to older persons due to a concomitant loss of muscle and bone density, which can exacerbate sarcopenia and increase the risk of fracture [79].

The weight loss interventions are similar for older and younger adults. Interventions for older adults should include an exercise program to preserve muscle mass because older adults tend to have an abridged muscle mass. Moreover, dieting promotes muscle loss in conjunction with fat loss. It has been noted that lifestyle interventions in the elderly population is able to yield up to a 10% weight loss in one year and is able to improve the symptoms of CVD and diabetes [80].

In older individuals with obesity, resistance exercise or aerobic plus resistance exercise reduces diet-induced muscle mass loss more than aerobic exercise alone. A study conducted in 2019 examined the long-term effects of weight loss combined with various exercise methods on myocellular quality and the sensitivity of muscle protein synthesis to food [81]. Older people with obesity were randomized to a weight-management program plus resistance, aerobic, or combination resistance and aerobic exercise, or to a control. At the baseline and six months, the participants underwent vastus lateralis biopsies and the results of the study showed that resistance and combination exercise had higher rates of muscle protein production than the control. Aerobic exercise, which saw a greater rise in inflammation and the expression of mitochondrial regulators, suffered a greater decrease in autophagy mediators than the combined exercise program. For enhancing muscle protein synthesis and myocellular quality in older individuals with obesity, combined aerobic and resistance training is superior to either modality used alone. This helps to preserve muscle mass while undergoing weight-loss therapy [81].

### 4.5. Bariatric Surgery

By limiting the amount of food the stomach can contain, bariatric surgery, also known as gastric sleeve or gastric bypass, causes weight loss by causing gastric restriction and nutritional malabsorption. The procedure offers several positive health effects, including a long-term weight loss of between 60 and 80 percent of extra weight, decreases in gut hormones, and dietary restrictions. Bariatric surgery seems to be the better option for treating obesity and the disorders it is associated with in individuals for whom conventional treatments for obesity, such as medications and behavioral and physical activities, have not been able to slow down the disease progression [82]. It would be reasonable to assume a greater risk of morbidity and mortality in elderly individuals who underwent surgery due to their lower physiological reserve and tissue fragility [83]. However, a recently published paper explored the outcomes of bariatric surgery in elderly individuals in a cohort study with three-year follow-up and found that following bariatric surgery, patients aged <65 and ≥65 exhibited comparable perioperative morbidity and mortality. Despite the fact that patients under 65 years old had superior medium-term outcomes overall, bariatric surgery was safe, produced significant weight loss, and improved comorbidities in the patients under 65 [84].

### 4.6. Bariatric Equipment

**Figure 3:**
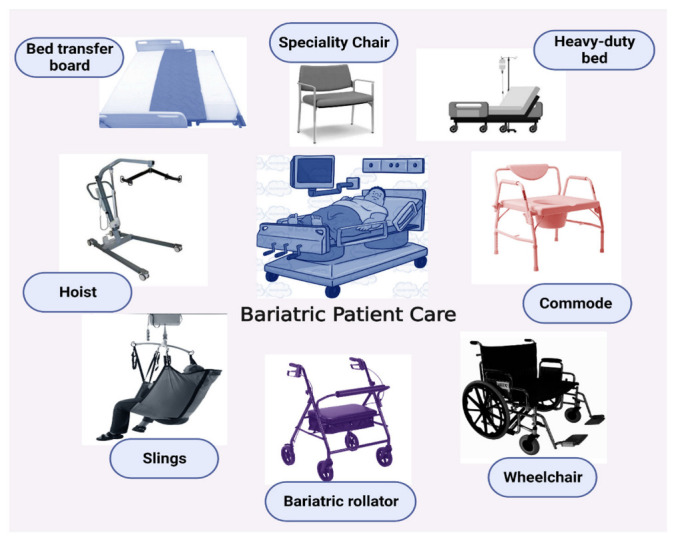
Individuals with morbid obesity typically require specialized bariatric equipment to perform their daily chores.

Bariatric equipment may be necessary to care for patients with morbid obesity (refer to Figure 3). Examples of bariatric equipment include the following:

Heavy-duty beds

1. Bariatric mattresses,
2. Hoists,
3. Slings,
4. Bed transfer boards,
5. Bariatric wheelchairs,
6. Bariatric rollators.

It is important to note that patients with a BMI of 40 or greater must use only specialized bariatric equipment designed to handle more than 350 pounds [85].

## 5. Caregivers for Individuals with Obesity

Individuals who provide care to people with chronic illnesses or disabling conditions by helping them with functional tasks of daily living are known as caregivers. Formal caregivers are paid to help people with chronic illnesses or disabling conditions (e.g., obesity). Formal caregivers may provide in-home care to provide: (1) bathing and grooming, (2) mobility assistance, (3) meal preparation, (4) grocery shopping assistance, and/or (5) other daily living tasks. Other examples of formal caregivers may include occupational therapists, physical therapists, nurses, and/or nursing assistants.

Caregivers are categorized as formal or informal. Family, friends, community members, and/or relatives are often informal caregivers who provide unpaid care for individuals with obesity. According to 2009 data from the state-based Behavioral Risk Factor Surveillance System at the Centers for Disease and Control (CDC), approximately 25% of U.S. adults 18 years of age and older had provided “informal or unpaid” care to a person with a long-term illness or disability in the past 30 days. The one-year value of this unpaid caregiver activity was approximately USD 450 billion dollars in 2009 [86].

**Figure 4:**
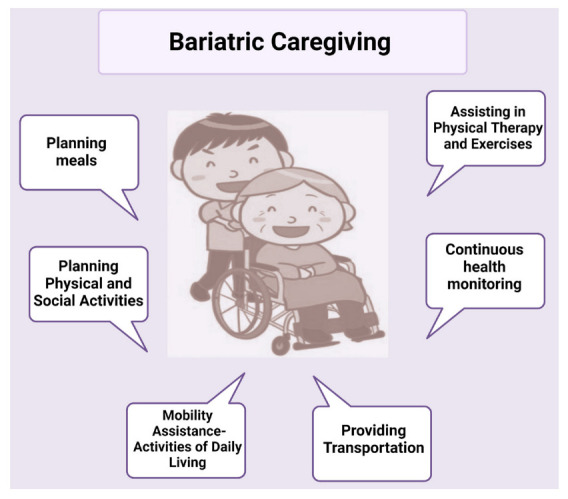
Caregiving responsibilities of a bariatric caregiver.

In patients with obesity, informal caregivers provide critical support to the patients—both mental and physical (refer to Figure 4). Caregivers’ attitudes towards controlled feeding can be both enabling and restrictive towards the feeding behaviors of the patient with obesity. In infants and children, it was seen that mothers (i.e., primary caregivers) with a flexible attitude and higher cognitive function were able to better support the nutritional needs required for obesity management [44].

### 5.1. Challenges Encountered by Family Caregivers

Individuals with obesity who are usually younger than normal weight individuals with disabilities may have less nonworking family members and may be less likely to seek a caregiver. This struggle by caregivers, particularly with physically challenging activities involving mobility or personal care, may lead to less accessible, qualified caregivers than an individual of normal weight.

One potential explanation for the link between obesity and a lesser quality of care at home is incapacitated adults. Individuals with obesity are more likely to be admitted to a nursing home and are more likely to fall. A study established that incapacitated adults with obesity of all ages experienced lower rates of paid help than adults without obesity; however, this data was elucidated by distinctions in younger people with obesity and the analyses did not inspect individual types of disability shortfalls [87]. Elderly individuals with obesity require more care than younger adults with obesity due to other concomitant conditions such as Alzheimer’s Disease (or other forms of dementia), sarcopenia, osteoporosis, diabetes mellitus, and several other chronic conditions associated with aging [77,87]. Caregivers for the elderly with obesity are then tasked with caring for challenging, high fall-risk patients with a multifaceted treatment plan with a mix of physical and pharmacological therapeutics [77].

Numerous older individuals in established countries are overweight or obese and the occurrence is snowballing, as more people who reach old age are already overweight. Obesity in old age is linked with an enlarged predisposition to diseases and a compromised quality of life [88]. There is a comparative upsurge in mortality in older than young adults and the body weight connected with the highest survival rises with progressing age. Caring for patients with obesity takes an increased amount of physical labor and time, and this workload is only exacerbated by increased age [89].

Family caregivers have many challenges in caring for individuals who have obesity. These include: (1) an increasing age of the caregiver population, (2) the risk of injury, and (3) access to care management tools. These challenges may cause caregivers to experience a significant amount of biological, psychosocial, and financial burden [90].

#### 5.1.1. A. Increasing Caregiver Age

Studies show that a decline in health of over half (53%) of caregivers compromises their ability to provide care for individuals with obesity. As the number of older Americans increases, so will the number of caregivers needed to provide care. The number of people 65 years old and older is expected to double between 2000 and 2030. It is expected that there will be 71 million people aged 65 years and older when all baby boomers are at least 65 years old in 2030. Currently, there are seven potential family caregivers per adult. By 2030, there will be only four potential family caregivers per adult [86].

#### 5.1.2. B. Risk of Caregiver Injury

Caregivers are challenged with the day-to-day care (e.g., mobility, skin care, and personal hygiene) of individuals with obesity. In the inpatient setting, patients with morbid obesity require a mean of 4.5 individuals to assist them with walking as opposed to 1.9 individuals for adults without obesity. Studies indicate that patients with obesity in nursing homes require a higher level of personnel to provide care.

Of caregivers, nurses and nursing assistants in the U.S. are reported as being at a high risk of suffering musculoskeletal injuries, while currently, 46% of nursing assistants have self-reported a musculoskeletal injury while moving patients, 40% have reported back injuries, and a reported 50% of nursing and support staff have considered quitting due to the risk of injury from physical stress [91]. This does not offer data or insight into the caregivers responsible for caring for a family member or a loved one that is not part of their primary occupation. Caregivers and healthcare workers face several challenges when attempting to mobilize these patients such as an unevenly distributed weight, varying levels of patient mobility and mental capacity, and uncooperative behaviors [91,92]. Additionally, typical patient handling techniques are ineffective and cannot be used by caregivers due to a patient’s weight and size [91].

### 5.2. Caregiver Management Tools

The caregivers of patients with obesity face unique challenges such as the lack of bariatric equipment at home, a lack of knowledge about nutrition, and access to formal weight loss programs. The presence of individual factors such as poverty may create a situation where accessing healthy food and/or a weight loss program are problematic [93]. The prejudice surrounding obesity may prevent individuals with obesity from requesting aid [94]. In addition, the caregivers of individuals with obesity may sabotage them from obtaining assistance because they feel that the severity of their condition does not warrant professional care [87]. There is a lack of clinical research and guidelines for the caregivers of patients with obesity, and specifically those who are growing older [95].

The guidelines for caregivers include risk management, which begins with an assessment completed by physicians and the healthcare team. This team examines and identifies capabilities, challenges, and all the health conditions associated with a patient to form the best treatment plan to aid caregivers [91]. The guidelines then focus on the following factors:

1. Availability of appropriate bariatric equipment and supplies,
2. Can the home or patient care location incorporate and fit this equipment?
3. Adequate walking space in hallways and doorways,
4. Adequate number of caregivers or nursing staff.

These guidelines are centered around families and patients having access and the financial means to a healthcare facility for patients with obesity [92]. Frequently, however, these patients are from a lower socio-economic background and do not have the means to afford such care or to access the necessary bariatric supplies. This poses a major challenge for patients and their caregivers and there are also new restrictions within formal clinic and hospital settings. A “no lift” policy has been implemented in numerous facilities, that instead requires staff to use machinery and other bariatric equipment for patient mobilization [91,92]. These restrictions, in part, have been initiated to prevent financial devastation due to paying for multiple employee injuries; however, these new restrictions could also further complicate the care for patients with extreme obesity, leading to an increased wait time for patients and an increased time commitment for the support staff.

## 6. Conclusions and Future Directions

Obesity is a global pandemic that affects millions of individuals and is one of the leading modifiable contributors of mortality and morbidity. Obesity, which presents as an increased accumulation of adiposity is a precursor to comorbidities such as diabetes, hypertension, CVD, certain types of cancers and others. Hispanic populations have a high incidence of obesity and tend to be more exposed to the obesogenic risk factors such as socio-economic conditions, a lack of physical activity, poor diet quality, a lack of nutritional knowledge, genetic predisposition, environmental triggers, and behavioral tendencies. As obesity progresses to severe and morbid states, individuals become increasing dependent on caregivers. Among Hispanics, this burden falls on family caregivers. Language barriers, a lack of awareness, financial hurdles, fear of the U.S. healthcare system, poor accessibility to affordable health care and an inability to obtain medical insurance, all contribute to a state of dire, life-threatening consequences for Hispanic patients with obesity. This in turn causes a serious physical and mental drain on their family caregivers. This vicious cycle then continues with little to no reform as indicated by the dearth of scientific literature in this area. Future research studies should focus on developing culturally-competent measurement tools to assess the burden of caregivers and to better understand the needs of specific populations. Moreover, clinical trials should be designed and implemented that focus on the dietary and lifestyle needs of at-risk minority groups such as Hispanic populations. Healthcare providers should work hand-in-hand with community stakeholders to alleviate the environmental, cultural and social-demographic factors that increase the risk of obesity among Hispanics. Additionally, community outreach and health promotion activities that are culturally-relevant and designed should be implemented, both at the grass roots level as well as on a larger scale.

## Figures and Tables

**Table 1:** Adult weight classification is based on the body mass index (BMI) range as determined by the World Health Organization (WHO) [5].

BMI Range	Weight Classification
<18.5	Underweight
18.5–24.9	Healthy Weight
25.0–29.9	Overweight
30.0–34.9	Class I Obesity
35.0–39.9	Class II Obesity
>40.0	Class III Obesity

## Data Availability

Not applicable.

## Abbreviations

Ad-36	Adenovirus-36
AgRP	Agoutirelated protein
BAT	Brown adipose tissue
BMI	Body mass index
CDC	Centers for Disease Control
CNS	Central nervous system
CR	Calorie restriction
CER	Continuous energy restriction
DNA	Deoxyribonucleic acid
CVD	Cardiovascular disease
GDP	Gross domestic product
NCD	Non-communicable diseases
NHANES	National Health and Nutrition Examination Survey
NPY	Neuropeptide-y
OAO	Overweight and obesity
POMC	Proopiomelanocortin
RMR	Resting metabolic rate
SES	Socio-economic status
U.S.	United States
WAT	White adipose tissue
